# Pathologist-initiated reflex testing for biomarkers in non-small-cell lung cancer: expert consensus on the rationale and considerations for implementation

**DOI:** 10.1016/j.esmoop.2023.101587

**Published:** 2023-06-23

**Authors:** J.R. Gosney, L. Paz-Ares, P. Jänne, K.M. Kerr, N.B. Leighl, M.D. Lozano, U. Malapelle, T. Mok, B.S. Sheffield, A. Tufman, I.I. Wistuba, S. Peters

**Affiliations:** 1 Department of Cellular Pathology, Royal Liverpool University Hospital, Liverpool, UK; 2 Hospital Universitario 12 de Octubre, H12O-CNIO Lung Cancer Unit, Ciberonc and Complutense University, Madrid, Spain; 3 Lowe Center for Thoracic Oncology, Dana-Farber Cancer Institute, Boston, USA; 4 School of Medicine and Dentistry, University of Aberdeen, Aberdeen, UK; 5 Princess Margaret Cancer Centre, Toronto, Canada; 6 Pathology, Universidad de Navarra-Clínica Universidad de Navarra, Pamplona, Spain; 7 Department of Public Health, University of Naples Federico II, Naples, Italy; 8 Department of Clinical Oncology, The Chinese University of Hong Kong, Hong Kong SAR, China; 9 Department of Pathology and Laboratory Medicine, William Osler Health System, Brampton, Canada; 10 Department of Internal Medicine V, Thoracic Oncology Centre Munich, Ludwig Maximilian University, Munich; 11 Comprehensive Pneumology Center Munich (CPC-M), Munich; 12 German Center for Lung Research (DZL), Munich, Germany; 13 Departments of Thoracic/Head and Neck Medical Oncology; 14 Translational Molecular Pathology, The University of Texas MD Anderson Cancer Center, Houston, USA; 15 Department of Oncology, Centre Hospitalier Universitaire Vaudois (CHUV), Lausanne, Switzerland

**Keywords:** reflex testing, non-small-cell lung cancer, NSCLC, predictive biomarkers, turnaround time, molecular pathology

## Abstract

Biomarker tests in lung cancer have been traditionally ordered by the treating oncologist upon confirmation of an appropriate pathological diagnosis. The delay this introduces prolongs yet further what is already a complex, multi-stage, pre-treatment pathway and delays the start of first-line systemic treatment, which is crucially informed by the results of such analysis. Reflex testing, in which the responsibility for testing for an agreed range of biomarkers lies with the pathologist, has been shown to standardise and expedite the process. Twelve experts discussed the rationale and considerations for implementing reflex testing as standard clinical practice.

## Highlights

• Complete and timely biomarker testing is crucial for selecting first-line systemic therapy for patients with non-small-cell lung cancer.
• Reflex testing is expeditious and standardises the ordering of biomarker tests to ensure more patients are tested.
• Recent clinical and technological advancements will help to overcome concerns of increased costs and limited reimbursement.
• Reflex testing must be governed by a protocol defined by the multidisciplinary team (MDT).
• MDT collaboration and engagement with policy makers are required to establish and maintain the reflex testing protocol.

## Introduction

The management and treatment of patients with non-small-cell lung cancer (NSCLC) have evolved rapidly over recent years owing to the progressive increase in the availability of different targeted therapies and novel immunotherapies, which have dramatically improved response rates and survival in patients with metastatic disease. The advent of such targeted therapies has made biomarker testing an essential prerequisite for guiding optimal first-line systemic therapy. More recently, the introduction of targeted therapy in the adjuvant setting has meant that biomarker testing is also becoming important for clinical decision making in early-stage disease. As the number of targeted therapies inexorably increases, so will the demand for timely clinical investigation of numerous biomarkers across multiple different genes. According to the recent international guidelines, treatment of metastatic non-squamous NSCLC should be guided by the genomic evaluation of mutations and fusions such as epidermal growth factor receptor (*EGFR*), anaplastic lymphoma kinase (*ALK*), ROS proto-oncogene 1 receptor tyrosine kinase (*ROS1*), v-raf murine sarcoma viral oncogene homolog B (*BRAF*), neurotrophic tyrosine receptor kinase (*NTRK*), rearranged during transfection (*RET*), Kirsten rat sarcoma viral oncogene homolog (*KRAS*) and MET proto-oncogene, receptor tyrosine kinase (*MET*), and by assessment of the expression of programmed death-ligand 1 (PD-L1) by immunohistochemistry (IHC).^1^, ^2^, ^3^, ^4^ Given that these tests are a fundamental requirement in the management of patients with NSCLC, reliable processes and methods are required to ensure they are carried out in every appropriate case and that the results are available to the treating oncologist in time to permit planning of treatment for every patient.

Traditionally, the ordering of biomarker tests has been a matter for the treating oncologist, who would consider which were necessary for each individual patient based on the histological diagnosis made by a pathologist and then order those tests. A major problem with this traditional, oncologist-led pathway is that it delays the initiation of complex and often lengthy testing procedures and, therefore, delays the availability of the information necessary to inform therapy.^5^^,^^6^ Because there is a risk of clinical deterioration while waiting for the results of biomarker tests, delays in test reporting can lead to the initiation of suboptimal therapy before determining complete biomarker status.^7^ Long test turnaround times may also deter clinicians from ordering biomarker tests.^8^^,^^9^ A retrospective review of 1203 patients with pulmonary adenocarcinoma reported that the percentage of tumours tested for *EGFR* mutation was 54%, *ALK* mutation 51%, *ROS1* mutation 43%, *BRAF* mutation 29%, *RET* mutation 17%, *MET* exon 14 skipping 15% and Erb-B2 receptor tyrosine kinase 2 (*ERBB2*) mutation 11%.^10^

Implementation of reflex testing is one potential way to reduce the time from diagnosis to the delivery of all clinically actionable biomarker results while also helping to ensure more patients are tested. A roundtable meeting of 12 experts with an even distribution of oncologists and pathologists was convened in May 2022 to discuss the prospect of reflex testing becoming the new standard in clinical practice. The present article presents a summary statement of the expert working group.

## The case for change—reflex testing, a new standard of care

Reflex testing is an approach to testing in which the pathologist handling the case is responsible for initiating and controlling testing for a set of prespecified biomarkers, agreed upon within the context of the multidisciplinary team (MDT), but without the need for a formal request from an oncologist.^11^ This approach was recommended in the earliest days of genomic profiling of NSCLC,^12^^,^^13^ and was established as standard practice in some centres at this time. It is still by no means universally practised, however, and the case for it continues to be made, as in a recent publication,^14^ which makes a strong argument for it from the point of view of seeking *EGFR* mutations in particular. The case for reflex testing, therefore, has clearly not yet been universally accepted.

Reflex testing allows biomarker testing to begin as soon as the pathological diagnosis is confirmed, rather than waiting until after the patient’s first post-biopsy appointment with the oncologist or after discussion by the MDT.^15^

Reflex testing has several attributes that make it the optimal approach for ordering biomarker tests in patients with NSCLC. Firstly, through the application of a protocol defined by the MDT, reflex testing ensures that all patients with NSCLC are guaranteed optimal care at a local level regarding the extent of biomarker evaluation. Unlike the traditional oncologist-led testing pathway, which depends on clinical consideration by the oncologist, a reflex testing protocol depends on testing ordered routinely immediately after pathological confirmation of the diagnosis. This means that the ordering process is systematic and bypasses complexities of clinical consideration that might otherwise influence the decision on which tests to request. As a result of this, fewer patients are overlooked for testing, creating a more systematic and equitable system. Additionally, several socioeconomic and racial factors might create significant disparities in the cancer journey, including and starting with biomarker testing.^16^ In real-world studies, the absolute number of patients being tested and, crucially, the overall mutation detection rate increased after the introduction of reflex testing.^17^, ^18^, ^19^, ^20^, ^21^ Reflex testing is expeditious, reducing both turnaround time and the time to optimal first-line systemic therapy.^6^^,^^17^^,^^22^

Accelerating the time to initiating first-line treatment is clinically important. Patients with untreated advanced NSCLC can deteriorate rapidly, becoming too ill for therapy or even dying before treatment is initiated.^23^ Expediting biomarker testing can help to minimise the risk of such situations significantly, while reducing the additional costs associated with delayed care.^24^ Reflex testing of patients with early-stage, resectable disease is now also vital. The data that continue to emerge from the ADAURA trial, for example,^25^ in which adjuvant therapy is applicable to even stage 1b disease, make clear the importance of seeking *EGFR* mutations as early as possible in either the resected tumour or, ideally, presurgical specimens if available, and targeted therapies are now becoming established in the neoadjuvant setting.

The expert working group also discussed how reflex testing facilitates and optimises tissue stewardship, a key factor for the successful completion of biomarker testing in NSCLC, where the amount of material available for testing is often very limited. The traditional two-stage diagnostic and testing pathway—in which initial assessment and diagnosis by a non-specialist pathologist in a community hospital is followed by referral to a specialist thoracic pathologist, usually in an academic centre, who continues the process—does not facilitate optimal handling of precious material.^26^ After adopting a reflex testing protocol, one study observed significantly fewer unsuccessful tests for *EGFR* mutations and *ALK* fusions (14% before adoption versus 4% after and 17% before adoption versus 3% after, respectively).^17^ In addition, optimising tissue stewardship and increasing successful analysis of the initial diagnostic specimen will inevitably reduce the unwelcome requirement for a second biopsy.

The expert working group recognised that one of the barriers to the adoption of reflex testing is concern over the potentially high cost, given that such testing is not generally stratified according to clinical features such as stage, smoking history and performance status, nor by imaging; such information is generally not available to the pathologist guiding the analysis. Inevitably, this means that there is potential for testing that might be deemed unnecessary or inappropriate. In the context of current targeted adjuvant therapy approaches, and the expectation that the use of targeted therapies before stage IV (including in the neoadjuvant setting) will continue to expand, the argument that reflex testing algorithms lead to over-testing in early-stage disease loses traction.

In addition, any potential financial disadvantage must be weighed against the advantages already outlined and also considered in the context of the overall cost of managing a patient with lung cancer. For a patient whose tumour has a genomic driver or a high level of PD-L1 expression rendering it responsive to immunotherapy, treatment might extend life for many months or even years, over which time the cost of this successful therapy is considerable. As more targets emerge and more drugs active against them are developed, survival will continue to lengthen. The cost of carrying out a range of predictive biomarker tests, especially if next-generation sequencing (NGS)-based and even if repeated, is already minute as a proportion of the overall cost, and will diminish increasingly as we move into the future.

Another counter to the challenge that reflex testing might incur unnecessary costs is the increasing availability of NGS, which is quickly becoming preferred over ‘single-gene’ polymerase chain reaction-based analysis for biomarker testing of patients with NSCLC.^27^ NGS is considered less time-consuming and financially less demanding than single-gene testing when a number of genomic alterations are being sought as is increasingly the case for NSCLC.^28^^,^^29^

Reimbursement of cost also impedes the adoption of reflex testing. Reimbursement practices differ by country and may be limited and/or constrained to specific requirements. For example, reimbursement may be predicated on documented disease stage, or it might be mandated that the order for a particular test or tests comes only from a treating physician. However, it might be possible to navigate this barrier within the MDT by, for example, agreeing on a ‘standing order’ (remaining disease stage agnostic) for a particular range of tests effectively ordered by the oncologist and instigated automatically by the pathologist, which might evolve over time.

Finally, the argument is sometimes made that the results of biomarker testing on an initial diagnostic specimen might be irrelevant as disease advances and the tumour evolves, developing new genomic alterations and changing its response to immune attack as evidenced by expression of PD-L1. The opinion of the group, however, is that this should be viewed not as an argument against testing the initial diagnostic specimen but as one for routine re-biopsy on progression. In the specific case of addictive oncogenic drivers, e.g. *EGFR* mutation and *ALK* fusion genes, these are almost always truncal and do not evolve during disease progression; therefore, the status of these markers, at least in the initial sample, is likely to be relevant to relapsed disease. Nonetheless, biopsy at progression is valuable for other reasons.

## Defining the protocol for reflex testing

Appropriate implementation of reflex testing must be governed by the availability of a protocol that clearly defines which diagnoses would drive the ordering of biomarker tests and which tests are to be ordered. The details of the protocol will be institution-specific and are a matter for agreement among the MDT, comprising representatives from a range of specialties including pathology, oncology, surgery and radiology.

The expert working group recommends a stage-agnostic reflex testing protocol, where all standard and evidence-based clinically actionable biomarkers are automatically ordered by the pathologist and tested, usually by a combination of NGS and IHC. This approach would ensure complete biomarker profiling, accelerate the time to results and be cost-effective compared with single-gene testing of multiple biomarkers.

Subtyping of NSCLC into squamous and non-squamous by pathologists has been traditional for many years and is embedded in various guidelines.^4^^,^^30^, ^31^, ^32^ It is widely used to guide which tumours should be analysed for which biomarkers and the search for mutations and fusions generally being confined to non-squamous tumours on the grounds that they are only very rarely drivers of squamous cell carcinoma.^33^, ^34^, ^35^ Such subtyping is possible on morphological grounds alone in ∼70% of tumours; IHC for thyroid transcription factor-1 and p40 should be employed to make the distinction in the remainder and should reduce the ‘NSCLC not otherwise specified’ rate to 10% or less.^36^^,^^37^ The additional IHC testing required for this distinction does contribute to attrition of the specimen, but this group of NSCLCs that are not easily subtyped may still harbour genomic drivers.^4^^,^^31^ When IHC is used in this context, pathologists should use only the minimum amount of tissue required to make the distinction.^36^

Ultimately, each institution needs to define its own protocol for reflex testing. Ideally, this should align with international and, where available, national testing guidelines, while considering the local situation regarding available tests and technology, treatment strategies and available resources.

## Guidance for implementing reflex testing

### MDT agreement on the concept of reflex testing and the decision to implement

Adopting reflex testing should be an institutional decision involving close communication between radiology, pulmonology, thoracic surgery, oncology and pathology.^38^ This is needed to ensure that all members of the MDT agree with the concept and that the three principles of reflex testing are followed: (i) testing of predefined biomarkers, (ii) initiated by the pathologist and (iii) upon an appropriate diagnosis. Clearly, the thoracic oncologist is likely to take the lead in discussion and decision making, but all members of the team should have a sense of engagement and feel that they have a role to play. Only after the MDT is convinced of the need to test reflexively can the subtleties of the protocol (discussed in the section ‘Defining the protocol for reflex testing’) be defined. It is important to remember that implementing reflex testing does not preclude traditional oncologist-led testing in cases not covered by the reflex testing protocol.

### Engage with institutional policy makers

The expert working group recommends broader collaboration between not only members of the MDT but also institutional policy makers such as hospital, regional and national administrations to understand the feasibility of the protocol that has been defined and agreed by the MDT in accordance with local circumstances. In the United States, for example, Centers for Medicare and Medicaid Services (CMS) actually requires that such testing be ordered by the treating clinician. As such, there is a need for engagement at the highest levels to encourage flexibility in the conditions for reimbursement in a particular country; it is important to ensure policy makers understand the limitations of the traditional ordering process and the rationale for reflex testing. Commercial laboratories should also be engaged as appropriate. A multidisciplinary-led presentation to hospital and other administrations referring to data in published literature about how reflex testing can improve the quality of care and reduce costs can facilitate rational and operational discussions. Showing the cost benefits in different clinical departments is essential in justifying a potentially increased budget within the laboratory. There is evidence available to help make the case for the improved efficiency and cost-effectiveness of reflex testing using NGS compared with the traditional way of ordering and carrying out biomarker tests.^6^^,^^17^, ^18^, ^19^, ^20^, ^21^^,^^23^^,^^24^^,^^26^^,^^27^ Ultimately, institutional policy makers need to be convinced that the proposed protocol for reflex testing is efficient and cost-effective, and engagement with payers is necessary to secure reimbursement.

### Establish roles and responsibilities between the referring pathologist, specialist thoracic pathologist and molecular pathologist/scientist

Once institutional policy makers have agreed to the protocol, it is important to define roles and responsibilities between the referring pathologist (who has responsibility for the earliest stewardship of the specimen and must be aware of the need for its judicious management), the specialist thoracic pathologist (who is best-placed to consider how to manage the need for genomic profiling with the requirement for other tests such as PD-L1 assessment by IHC) and the molecular pathologist or scientist (who is not always in-house, but may be part of a centralised, regional or supra-regional molecular testing facility).

The initial step in any reflex testing protocol is making the pathological diagnosis, and this responsibility is likely to remain with the referring pathologist. Although diagnosis of tumour (sub)-type is the priority, it is imperative that the referring pathologist does not waste tissue by carrying out unnecessary diagnostic IHC tests. If there is any doubt about specimen sufficiency for complete biomarker testing, the expert working group recommends that an assessment of sample adequacy should be made by the pathologist in, or in discussion with, the laboratory carrying out the molecular tests because sample requirements differ depending on the testing modality and strategy used. Establishing procedures for quality control is also crucial.

It is imperative to regularly check whether the reflex testing protocol is being implemented effectively and having the desired impact on patient care. The expert working group recommends establishing a process for measuring protocol performance such as tracking the percentage or number of patients who present to their first oncology visit with a complete set of biomarkers available.

### Maintenance of the protocol

Given that change in diagnostics and profiling of lung cancer is fast-paced and continually evolving, the expert working group recommends frequent evaluation of the reflex testing protocol to ensure it complies with the latest local and international guidelines and reimbursement conditions.

## Conclusion

Biomarker testing is critical to appropriately guide the selection of systemic therapy for NSCLC, in the adjuvant setting as well as in patients with metastatic disease. However, patients with lung cancer are often inadequately tested for biomarkers due to non-standardised ordering practices and long turnaround times associated with traditional oncologist-led testing. According to the expert working group, reflex testing should be regarded as the optimal strategy for biomarker testing in NSCLC and this is also the recommendation of recent international guidelines.^2^

The practice of reflex testing must be guided by the availability of and adherence to a protocol, defined and agreed by the MDT, in accordance with local circumstances. Where possible, comprehensive reflex biomarker testing, ideally via NGS and IHC, in all patients with an appropriate NSCLC diagnosis, and irrespective of disease stage, is recommended.
